# Fatty Acid Composition, Antioxidant, and *in vitro* Anti-inflammatory Activity of Five Cold-Pressed *Prunus* Seed Oils, and Their Anti-biofilm Effect Against Pathogenic Bacteria

**DOI:** 10.3389/fnut.2021.775751

**Published:** 2021-11-16

**Authors:** Florinda Fratianni, Antonio d'Acierno, Maria Neve Ombra, Giuseppe Amato, Vincenzo De Feo, Juan Fernando Ayala-Zavala, Raffaele Coppola, Filomena Nazzaro

**Affiliations:** ^1^Institute of Food Science, National Research Council of Italy (CNR), Avellino, Italy; ^2^Department of Pharmacy, University of Salerno, Fisciano, Italy; ^3^Centro de Investigacion en Alimentacion y Desarrollo, Hermosillo, Mexico; ^4^Department of Agricultural, Environmental and Food Sciences, University of Molise, Campobasso, Italy

**Keywords:** *Prunus*, fatty acids, antioxidant, anti-inflammatory, biofilm

## Abstract

**Background/Aim:** Sweet almond (*Prunus amygdalus dulcis*) oil is one of the most famous cold-pressed seed oils. However, other species of *Prunus* can provide oils with healthy properties. We analyzed the fatty acid (FA) composition, as well as the antioxidant, the *in vitro* anti-inflammatory properties, and the antibiofilm activity of five commercial vegetable cold-pressed seed oils of apricot, peach, plum, cherry, and black cherry.

**Methods:** Gas Chromatography-Mass Spectrometry was performed for the analysis of FAs The antioxidant property of the oils was carried using different tests [2, 2-diphenyl-1-picrylhydrazyl (DPPH assay)], Ferric Reducing Antioxidant Power (FRAP), and the 2, 20 -azino-bis (3-ethylbenzothiazoline-6-sulfonic acid) (ABTS·+). The denaturation assay performed on bovine serum albumin (BSA) was used to evaluate the *in vitro* anti-inflammatory activity. The anti-biofilm activity was assessed using five pathogenic strains, namely, *Acinetobacter baumannii, Escherichia coli, Listeria monocytogenes, Pseudomonas aeruginosa*, and *Staphylococcus aureus*, through the crystal violet test and the 3-(4,5-dimethylthiazol-2-yl)−2,5-diphenyltetrazolium bromide (MTT), used to evaluate the metabolism of the microbial cells present within the biofilm.

**Results:** Oleic acid and linoleic acids were the most abundant FAs. Black cherry seed oil exhibited the best antioxidant activity, but in general, the amount of oil needed to inhibit the activity of 1 ml of DPPH assay at 50% did not exceed 10 μg. The extract concentration for the 50% inhibition of the denaturation of the protein (IC50) did not exceed 4.4 μg. Linoleic and stearic acids affected the antioxidant activity of the oils; oleic acid, linolenic, and palmitoleic acids exhibited beneficial effects in preserving the BSA denaturation, as shown by the correlation data. The oils were able to inhibit the biofilm formation of the pathogens (up to 71.40% of inhibition) as well as act against their mature biofilm, although with different strengths, with values up to 61.54%. Concurrently, they also acted on the pathogen metabolism.

**Conclusion:** The oils represent a valuable source of some healthy FAs. They showed potential antioxidant and anti-inflammatory *in vitro* activity, in addition, their potential effect on the biofilm can offer important ideas for research and reflection on their use as functional foods and/or ingredients.

## Introduction

Seed oils are oils obtained from fruits or seeds of plants and trees other than the olive tree. Such oils have a remarkable versatility, being able to be used in the food sector first, but finding applications in the cosmetic, chemical, and pharmaceutical industries (for example as a vehicle for some drugs, in the preparation of infusions for parenteral nutrition, etc.). In addition to the olive tree, many other plant species have seeds (70%) or fruits (30%) with an oil content that makes extraction convenient. The extraction of vegetable oils can mainly take place in two ways: with mechanical procedures at temperatures (generally below 27°C), which maintain the characteristics of the oil unchanged, cold pressing, or refining by processes that involve the use of chemical processes and solvents that cause chemical and physical transformations of the oil. Cold pressing is not very profitable as it only allows 50% of the oil contained in the seeds to be obtained, but it permits obtaining oils with a high nutritional profile, unlike chemical extraction, which is more profitable but causes the loss of vitamins and other nutrients. The edible seed oil contains saturated (SFA), monounsaturated (MUFA), and polyunsaturated (PUFA) fatty acids (FAs), as well as polyphenols, tocopherols, and tocotrienols, lignans, phytosterols, triterpenes, carotenoids, and chlorophylls (1). Natural bioactive compounds, extracts, and oils from vegetables, fruits, seeds, and medicinal plants show well-ascertained robust health-promoting potentials that could act against different diseases. Pressed seed oils can play an important nutritional role in supporting cell functions, allowing the absorption of fat-soluble vitamins. In addition, they can contribute to the maintenance of tissues and the balance of the nervous tissue, as well as maintaining an adequate energy deposit and protecting the body from the cold, helping it to maintain the temperature around 37°C. From a health point of view, as it has now been widely ascertained that oils with a content of unsaturated fats, essentially monounsaturated, alpha-tocopherol, and antioxidants, can contribute to counteracting the inflammatory processes at the base of various degenerative diseases, cancer, and cardiovascular diseases. The modern diet reserves a large slice of fats, mainly saturated, representing 42% of the total daily calories. According to nutritionists, however, the percentage of lipids should be 25%, which includes both the fats added to foods and those contained in foods, therefore, in the case of a 2,000 calories diet, about 60 g per day. The WHO recommends the daily intake of omega 6 and omega 3 in a ratio of 4: 1 to 5: 1 (5% omega 6 and 1% omega 3). Thus, the demand for natural phytochemicals significantly strengthened, and consumers are in quest of finding natural products for healthy lifestyles (2). Natural products are also useful to develop new anti-inflammatory agents (3). The interest in cold-pressed oils increased since the cold-pressing process involves no heat, no chemical treatments, and no refining process. This allows for the maintenance of a high level of bioactive phytochemicals (4). Fruit seeds oils stimulated great interest because they are edible oils with a high degree of antioxidant radical scavenging properties and a broad spectrum of antimicrobial activity (5–7). Amongst its multiple functional properties, the effect of the seed oils against the growth and surface attachment ability of pathogens, prodromal to the formation of biofilms, is not so widely studied. Biofilm formation is a self-protective mechanism exhibited by bacteria that aggregate to create a complex structure to resist severe environmental conditions. This causes a rise of their surface attachment ability and a higher population density, with the production of extracellular polymeric substances and a series of chemical-physical and metabolic processes resulting in an augment of their pathogenic features (8, 9), including their resistance to the conventional antimicrobial agents and phagocytosis. In such a way, it becomes more difficult to eradicate the biofilm from the living hosts (10). The genus *Prunus* originating from Asia belongs to the Rosaceae family, Amygdaloideae subfamily (11). It includes about 430 species of deciduous or evergreen trees and shrubs naturally widespread along all temperate regions. The fruit is a fleshy drupe (“prune”), but unlike almonds where the seed is consumed, the edible part in most stone fruits includes the mesocarp and/or the exocarp. All *Prunus* species are highly appreciated by consumers; they are studied for their nutritional and bioactive properties positive to human health (12, 13). Such peculiarities are linked to preventing different diseases and disorders, including age-related declines (14, 15). The seed also contains nutrients that support growth until the developing seedling becomes autotrophic and establishes the next generation (16). The most famous cold-pressed oil belonging to this genus is the cold-pressed almond oil (*Prunus amygdalus*), which has well-known positive properties on human health (17). The available literature reports the traditional use of seed oils from some species from the Rosaceae family for therapeutic purposes, used in some countries for their anti-inflammatory properties (18).

Different studies reported the chemical and biochemical characteristics of the oils obtained from the seeds of the *Prunus* species, such as *P. armeniaca, P. mahaleb, P. dulcis* (19, 20), as well as from their health potentialities (21, 22). While taking the cost into account, consumers are heading toward the consumption of health products obtained with sustainable methods. The food industry has acknowledged these needs. Thus, in recent years, different types of seed oils other than the usual corn, sunflower, etc. were introduced to the market. The cold-pressure extraction allows the nutritional and health properties of such products to be more effectively maintained. The coronavirus disease (COVID) pandemic led many people to buy food online. Online research has revealed that numerous products are present on the virtual market, to the most unknown, which could be potentially healthy. We focused our attention on the cold-pressure seeds oils of different species of *Prunus*. From a purely scientific point of view, there is very little information on the FA composition of these oils, nor is much known about their antioxidant and anti-inflammatory properties. On the other hand, the capacity of these oils to inhibit the formation of bacterial biofilms and their maturation -prodromal events of increased pathogenicity and resistance of bacteria to conventional antibiotics- is scarcely established. The greater knowledge of their health properties would allow their greater use, in such a way as to not simply constitute a waste product and an environmental management problem. This would simultaneously result in an economic advantage for the industry; concurrently, consumers would thus have a larger basket of health products available without a large economic sacrifice. Our work has tried to fill this gap by evaluating some health characteristics, namely the FA composition, the antioxidant and *in vitro* anti-inflammatory properties, as well as the biofilm-inhibitory activity exhibited by five commercial cold-pressed seed oils of apricot, peach, cherry, plum, and black cherry oils.

## Materials and Methods

### Plant Material

Five commercial cold-pressed seed oils of apricot (*P. armeniaca* L.), peach [*P. persica* (L.) Batsch], cherry [*P. avium* [(L.) L.], plum (*P. domestica* L.), and black cherry (*P. cerasus* L.) were bought in a local market. As indicated by the manufacturers, the seeds were from cultivated plants, and were mechanically cold-pressed, avoiding the use of solvents. Samples were stored at 4°C avoiding exposure to light until the biochemical and microbial analysis.

### Biochemical Analysis

#### Determination of FA Profile

Fatty acid methyl esters (FAMEs) were obtained by transmethylation as previously described (23). Chromatographic separation was carried out using an HP-5MS capillary column (30 mm, 0.25 mm, and 0.25 μm) and helium as carrier gas (1 mL/min). The FAMEs injection (1 μl, 10% in CH2Cl2, v/v) was done in split mode (50:1). The injector temperature was 250°C, whereas the detector temperatures were 280 °C and 180°C for Flame Ionization Detector (FID) and Mass Spectrometry (MS), respectively. For the gas chromatography-mass spectrometry (GC-MS) analysis, the ionization voltage, the electron multiplier, and the ion source temperature were set at 70 eV, 900 V, and 230°C, respectively. The following elution program was used: 220°C for 6 min increased to 270°C at 3°C/min and held at 270°C for 4 min. The compounds were identified by calculating their Kovats retention index with respect to the reference standard. The analyses were run in triplicate and the values reported are the mean ± SD of three experiments.

#### Extraction for Antioxidant Assays

For the assessment of the different tests of antioxidant activity, the oils were mixed with acetone (Sigma, Milano, Italy) (1:1 v: v) and left for 1 h at room temperature. Then, methanol-HCl 1% was added (1:2 v: v). The samples were then centrifuged at 13,000 rpm for 5 min, recovering a supernatant. The pellet was treated again with the solution methanol-HCl 1% and centrifuged again. The two supernatant were pooled and used for the analysis.

#### 2,2-Diphenyl-1-Picrylhydrazyl (DPPH) Test

The free-radical scavenging activity of the *Prunus* cold-pressed seed oils was measured by using the stable radical DPPH (24). The analysis was performed in microplates, by adding 7.5 μl of the sample [previously diluted 1:1vol/vol in Dimethyl sulfoxide (DMSO)] to 303 μl of a methanol solution of DPPH (153 mM). Then, the absorbance was measured in a UV-Vis spectrophotometer (Cary Varian, Milano, Italy). The absorbance of the DPPH radical without the potential antioxidant, i.e., the control, was measured as a basis. The inhibition of the free radical by DPPH in percent (I%) was calculated through the following ways: I% [(Ablank – A sample/Ablank)] × 100, where Ablank is the absorbance of the control reaction (containing all reagents except the test compound), and A sample is the absorbance of the test compound read at 517 nm until 60 min. In addition, we calculated the EC50 value, referring to the number of samples need to inhibit the activity of 1 ml of DPPH at 50%. All the tests were carried out in triplicate.

#### Ferric Reducing Antioxidant Power Assay

*In vitro*, the antioxidant power of the *Prunus* cold-pressed seed oils was determined using FRAP assay according to Benzie and Strain (25). Briefly, 10 μl of the sample were added to a total volume of 1 ml. The antioxidant power was calculated as the difference in absorbance at 593 nm between the reading at 6 min and the reading at 0 min. These values were related to a standard curve made with a pure solution of quercetin. The results were expressed as the mg of quercetin equivalent (QE)/ml.

#### 2, 20 -Azino-Bis (3-Ethylbenzothiazoline-6-Sulfonic Acid) Test

The ABTS test on the *Prunus* cold-pressed seed oils was carried out following the method of Re et al. (26). The antioxidant standard used was 6-hydroxy 2,5,7,8-tetramethylchroman-2- carboxylic acid (Trolox, Sigma Aldrich, Milano, Italy). Trolox (2.5 mM) was prepared in methanol and used as a stock standard. The working standards were prepared daily and diluted with methanol. The ABTS, 2,2'-azinobis(3-ethylbenzothiazoline-6-sulfonic acid) diammonium salt, and potassium persulfate (di-potassium peroxdisulfate), and HPLC grade methanol were all obtained from Sigma-Aldrich, Italy. The ABTS and potassium persulfate were dissolved in distilled water to a final concentration of 7 and 2.45 mM, respectively. These two solutions were mixed and the mixture was allowed to stand in the dark at room temperature for 16 h before use to produce ABTS radical (ABTS·+). The ABTS radical solution was diluted with distilled water to an absorbance of 1.00 at 734 nm. The samples (final concentrations 0.0001–0.01 mg/ml) or Trolox standards (final concentration 0–20 mM) were added to the diluted ABTS·+ solution; the absorbance reading was taken 6 min after mixing (Cary Varian, Milano, Italy). The results were expressed as mg Trolox equivalent antioxidant capacity (TEAC) of samples. All determinations were carried out in triplicate. The data of the DPPH test were reported as EC50 (μg) representing the amount of the oils needed to inhibit the activity of 1 ml of the stable radical DPPH at 50% and as inhibition percentage; for the FRAP test as the equivalent of standard quercetin (mg) (QE)/g; for the ABTS test: as mg Trolox Equivalent (TE) /g of oil.

### *In vitro* Evaluation of the Potential Anti-inflammatory Activity

The *in vitro* anti-inflammatory activity of the oils was evaluated by using the inhibition of serum bovine albumin denaturation technique. The protocols reported in the literature (27, 28) were modified as follows: a stock solution of 0.5% (w/v) bovine serum albumin (BSA), 96% purity (Sigma, Milano, Italy) was prepared in a 0.05 M Tris-phosphate buffer saline solution, of which the pH was adjusted to 6.5 with glacial acetic acid. To study the anti-inflammatory activity, the reaction mixture (5 ml) comprised 0.2 mL of BSA, 2.8 ml of phosphate tris-phosphate buffer saline solution, and 2 ml of varying amounts (5-10-20-30 μg) of oils dissolved in methanol. The control consisted of 1 ml of BSA with 10 μl of methanol. The samples were then heated to 72°C for 5 min and cooled down. The absorbance was calculated at 660 nm by using a UV spectrophotometer (Cary Varian, Milano, Italy). The extract concentration for the 50% inhibition (IC50) of the BSA denaturation was determined with respect to the control and using diclofenac sodium (1 mg/ml) as the positive control.

### Antibacterial Properties of the Oils

#### Microorganisms and Culture Conditions

Gram-negative *Acinetobacter baumannii (*ATCC 19606), *Pseudomonas aeruginosa (*DSM 50071), *Escherichia coli (*DSM 8579), Gram-positive *Listeria monocytogenes* (ATCC 7644), and *Staphylococcus aureus* subsp. *aureus* (ATCC 25923) were used as test bacterial strains. The bacteria were cultured in Luria Broth for 18 h at 37°C (35°C in the case of *A. baumannii*) and 80 rpm (Corning LSE, Pisa, Italy) before the microbial analysis.

#### Minimal Inhibitory Concentration

We evaluated the MIC of each oil through the resazurin microtiter-plate assay (29). Multiwell plates were prepared in triplicate, and then they were incubated at 37°C for 24 h. *Acinetobacter baumannii* was grown at 35°C at the same conditions. The lowest concentration at which a color change occurred (from dark purple to colorless) revealed the MIC value of each oil.

#### Biofilm Inhibitory Action of the Oils

The capacity of the oils to affect the biofilm formation by the pathogenic bacteria was evaluated following the method of Caputo et al. (30) in flat-bottomed 96-well microtiter plates. The overnight bacterial cultures were adjusted to 0.5 McFarland (1.5 × 10^7^ cells/ml Densitometer cell density turbidity 0.3–15.0 McFarland, CAMLAB, Cambridge, United Kingdom) with fresh culture broth before the test. Ten microliters of the diluted cultures was distributed in each well, 9 μg/ml and 18 μg/ml of each sample- and Luria-Bertani broth was added, to have a final volume of 250 μl/well. Then, the microplates were fully covered with parafilm tape, with the aim to avoid the evaporation of the material present in the wells, and incubated for 48 h at 37°C (*A. baumannii* was incubated at 35°C). Planktonic cells were removed, and the attached cells were gently washed twice with sterile phosphate-buffered saline PBS. Two hundred microliters of methanol was added to each well and retained for 15 min to fix the sessile cells. The methanol was discarded, and each plate was left for the samples to dry. The staining of the adhered cells was performed with 200 μl of 2% w/v crystal violet solution added to each well and discarded after 20 min. The wells were lightly washed with sterile PBS and left to dry. Two hundred microliters of glacial acetic acid 20% w/v was added to let the release of the bound dye. The absorbance was measured at λ = 540 nm (Cary Varian, Milano, Italy). The percent value of the adhesion was calculated with respect to the control (cells grown without the presence of the samples, for whose we assumed an inhibition rate = 0%). Triplicate tests were done, and the average results were taken for reproducibility.

#### The Action of the Oils on Mature Bacterial Biofilm

The overnight bacterial cultures were adjusted to 0.5 McFarland with fresh Luria Bertani culture broth; 10 μl of the cultures was added in flat-bottomed 96-well microtiter plates, to have a final volume of 250 μl/well. Then, microplates were fully covered with parafilm tape, to avoid the evaporation of the material present in the wells, and incubated at 37°C (*A. baumannii* was incubated at 35°C). After 24 h of bacterial growth, the planktonic cells were removed, and the two concentrations of the oils, 9 μg/ml and 18 μg/ml of each sample- and Luria-Bertani broth were added, to have a final volume of 250 μl/well. After another 24 h of incubation, the sequential steps of the experiment, including the calculation of the percent value of inhibition compared with the untreated bacteria, were performed as described above.

#### Inhibition of Cell Metabolic Activity Within the Biofilm

The effect of two concentrations, the 9 and 18 μg/ml of the oils added at the beginning of the bacterial growth and after 24 h of incubation- was also evaluated on the metabolic activity of the bacterial cells through the 3-(4,5-dimethylthiazol-2-yl)-2,5-diphenyltetrazolium bromide (MTT) colorimetric method (30, 31) by adding the oils at the beginning of the experiment and after 24 h. After 48 h total of incubation, the bacterial suspension, representing the planktonic cells, was removed and 150 μl of PBS and 30 μl of 0.3% of MTT (Sigma, Milano, Italy) were added, keeping the microplates at 37°C (except than *A. baumannii*, incubated at 35°C). After 2 h, the MTT solution was removed and two washing steps were performed with 200 μl of sterile physiological solution. Then, 200 μl of DMSO was added to let the dissolution of the formazan crystals that were measured at λ = 570 nm (Cary Varian, Milano, Italy) after 2 h.

### Statistical Analysis

Data were expressed as average ± SD of triplicate measurements. The analysis also correlated the values of the antioxidant and anti-inflammatory activity of the oils with the FA composition, using the free software environment for statistical computing and graphics R (https://www.r-project.org/). The values of the antibacterial tests were expressed as the mean ±SD and statistically analyzed using a two-way ANOVA followed by Dunnett's multiple comparison test, at the significance level of *p* < 0.05, using GraphPad Prism 6.0 (GraphPad Software, Inc., San Diego, California, United States) and MATLAB software (Mathworks, Massachusetts, United States).

## Results and Discussion

Table 1 reports the FA profile of the five oils. As previously observed by Lazos (32), the oils exhibited a high percentage of unsaturated FAs, ranging from 81.4% (black cherry) to 91.7% (plum) oil, with oleic acid (39.4–76.3%) and linoleic acid (15.1–46.2%) being the dominants. Oleic acid was the main constituent in the FAs of all the studied oils except for cherry oil, where linoleic acid constituted the main component. The SFAs ranged from 6.0% in the apricot oil until 16.3% in the black cherry, which—if compared to the cherry oil—showed a 3.5 higher content of SFAs. In addition, palmitic acid was the dominant SFA ranging between 4.9% (in the apricot oil) and 9.2% (in the black cherry oil). Our results indicated that the oils of cherry and peach exhibited similar percentages of saturated (6.0 and 6.5%, respectively), and unsaturated (90.6 and 91.7%, respectively), FAs. Likewise, we observed a similar trend between plum oil and peach oil that contained 12.8–11.6% of SFAs, and 85.9 and 85.2% of unsaturated FAs, respectively. The FA profile of the oils confirmed the available data present in the literature for apricot (33), black cherry, plum (34), cherry (35), and peach (36). The ratio of the unsaturated/saturated FAs present in the oils of cherry, apricot, black cherry, plum, and peach was 0.15, 0.07, 0.20, 0.07, and 0.14, respectively. The high prevalence of unsaturated FAs is thus of great value for their beneficial effects (37).

**Table 1:** Fatty acid composition of the cold-pressed seed oils of apricot, cherry, peach, plum, and black cherry^*^.

	**Apricot oil (%)**	**Cherry oil (%)**	**Peach oil (%)**	**Plum oil (%)**	**Black cherry oil (%)**
Palmitic acid (C16:0)	4.9^c^ ± 0.5	7.6^c^ ± 1.1	8.1^c^ ± 0.4	6.3^c^ ± 0.9	9.2^c^ ± 0.8
Palmitoleic acid (C16:1)	0.7^e^ ± 0.1	0.2^e^ ± 0.0	0.5^e^ ± 0.0	0.1^d^ ± 0.0	0.2^e^ ± 0.0
Stearic acid (C18:0)	1.1^d^ ± 0.1	5.2^d^ ± 0.7	3.5^d^± 0.2	0.2^d^ ± 0.0	7.1^d^ ± 0.7
Oleic acid (C18:1)	65.2^a^ ± 1.2	39.4^b^ ± 1.2	65.1^a^ ± 1.2	76.3^a^ ± 1.6	43.9^a^ ± 1.3
Linoleic acid (C18:2)	24.5^b^ ± 0.9	46.2^a^ ± 2.0	19.1^b^ ± 0.7	15.1^b^ ± 0.8	37.2^b^ ± 1.0
Linolenic acid (C18:3)	0.2^e^ ± 0.0	0.1^e^ ± 0.1	0.5^e^ ± 0.1	0.2^d^ ± 0.0	0.1^e^ ± 0.0
Total	96.6	98.7	96.8	98.2	97.7
Saturated fatty acids	6.0	12.8	11.6	6.5	16.3
Unsaturated fatty acids	90.6	85.9	85.2	91.7	81.4
Ratio saturated/unsaturated	0.10	0.15	0.14	0.10	0.20

* *The data are expressed as percentages. The experiments were performed in triplicate and herein is reported the average (± SD). According to two-way ANOVA (a, b, c, d, e) values with the same letters within a row are statistically similar (P < 0.05)*.

### Antioxidant Activity

Many papers on the functionality in the literature are related to apricot and almonds, which exhibit liver and heart-protective, antioxidant, and anti-inflammatory effects (22). Supplementation with apricot seed oil could trigger a significant augment in the activity of antioxidant enzymes (glutathione peroxidase and catalase) (38). Thus, due to their appreciable protein content, *P*.kernels (after the removal of toxic substances) could represent a promising good source of food or a nutritionally balanced and completely safe ingredient to incorporate in food products (39). However, it can be said that some of the “toxic” compounds present in the kernel of *Prunus* species, such as amygdalin, in quantity, can have their therapeutic effects, for example on digestive processes and small properties; in some cases, they have even been used in the treatment of cancer (40, 41). Furthermore, the toxicological effect of an oral administration of cherry seed extracts did not result in the onset of adverse health effects, keeping the kidney and liver functions intact (42). Starting from such considerations made based on existing literature, we intended to evaluate the *in vitro* antioxidant properties of apricot, cherry, peach, plum, and black cherry seed oils, deliberately of commercial origin, therefore available to every consumer. Results are shown in Table 2. The antioxidant property of the oils was assessed using DPPH and ABTS radical scavenging capacity assay and FRAP assays, all performed three times to test their reproducibility and all performed to have a wider scenario about the antioxidant activity of the oils. The DPPH test allowed the quantification of the reducing capacity of the substance under examination whether it acts with hydrogen transfer or electron transfer, but generally, the limits of this analytical technique are in the presence of large sterically bulky molecules, which are unable to react with the reactive part of the radical. The FRAP test allowed the evaluation of only the reducing capacity through electron transfer, completely ignoring the action of antioxidants that act by hydrogen transfer, therefore it does not allow the measurement of the contribution of molecules, such as thiols and proteins, which also play a different antioxidant role fundamental in biological fluids. The ABTS test gave the opportunity of measuring both hydrophilic and lipophilic antioxidants over a wide pH range. Through the DPPH test, we found evidence that all the oils exhibited excellent antioxidant quality, with EC50 values lower than 10.01 μg (63%). The black cherry and cherry seed oils exhibited the best antioxidant activity, with the EC50 values equal to 7.28 μg (84%) and 7.68 μg (84%), respectively. The antioxidant activity exhibited by the five oils resulted higher than that showed by the oil of *P. serotine* (43). The FRAP test confirmed the good antioxidant capacity of the oils (r DPPH-FRAP = −0.82). The antioxidant capacity of the black cherry seed oil estimated by the FRAP assay was the highest, being higher than that of the cherry seed oil by 30%, and even double that of the other three oils. The ABTS test substantiated the excellence of the oils as antioxidant agents (r ABTS-DPPH = −0.87). Once again, the black cherry exhibited the best antioxidant behavior (0.377 TE mg/g); the other oils, except apricot, showed similar performances. In each case, our results indicated an excellent antioxidant activity, even higher than that found by Csakvari et al. in six Romanian varieties of almond seed oils, which values ranged between 1.35 mg/L TEAC and 1.54 mg/L TEAC (44). The activity evaluated through the FRAP test indicated that the apricot oil exhibited an antioxidant strength inferior to that observed by Stryjecka et al. (45) in the oil of five cultivars of *P. armeniaca* grown in Poland, but higher compared with that analyzed by Kostadinović Veličkovska et al. (46). The peach oil exhibited a good antioxidant activity too. The DPPH scavenging activity (74%) was completely comparable, for example, to that found in the peels and pulp of different varieties of peach which varied from 70.8 to 80.9 and 66.8 to 76.5% in peels, and 51.9–60.1 and 43.4–49.1% in pulps (47). Moreover, other by-products derived from the cultivation of *Prunus* species, such as leaves, are a rich source of biomolecules with antioxidant activity (48). This confirmed that the kernel of peach, as well as those of the other *Prunus* species, could undoubtedly represent a further source of bioactive components, meaning that this species could be exploited as much as possible. These oils could be good candidates in the search for natural, effective substances with an antioxidant activity completely comparable if not superior to that exhibited by synthetic additives (49). The correlation analysis (Table 3) showed that stearic acid affected the antioxidant activity more than the others did (*r* = −0.94). The correlation ranged between *r* = 0.82 and *r* = 0.88, considering the other tests of antioxidant activity carried out. Saturated stearic acid does not increase the serum cholesterol level nor it is atherogenic (50) and, in our experiments, its concentration was not among the highest, resulting in between 0.2 and 7.1%. Palmitic and linoleic acid were the other two FAs that beneficially acted on the antioxidant activity (*r* = −0.85 and −0.81, respectively). The presence of linoleic acid-which resulted as one of the most abundant FAs present in the five oil- reduces cholesterol, protects against ischemic stroke, and exercises helpful effects in diminishing heart disease (51). Palmitic acid is associated with raising total cholesterol levels in the plasma, but its effect can be variable-both toward raising low-density lipoproteins and improving the amount of beneficial high-density lipoproteins (50). An increase in high-density lipoprotein (HDL) cholesterol helps to fight against heart disease and stroke (52). Therefore, the presence of these FAs improves the therapeutic value of these seed oils.

**Table 2:** Anti-oxidant activity exhibited by the cold-pressed seed oils of apricot, peach, cherry, black cherry, and plum, assessed through the 2,2-diphenyl-1-picrylhydrazyl (DPPH), Ferric Reducing Antioxidant Power (FRAP), and 2, 20-azino-bis (3-ethylbenzothiazoline-6-sulfonic acid) (ABTS) tests.

	**DPPH (EC50) μg/ml DPPH**	**DPPH (%)**	**FRAP mg (QE)/gr**	**ABTS TEAC mg (TE)/gr**
Apricot	10.01 ± 1.48	63	0.128 ± 0.09	0.262 (± 0.035)
Peach	9.04 ± 0.08	74	0.120 ± 0.03	0.293 (± 0.027)
Cherry	7.68 ± 0.08	84	0.178 ± 0.14	0.297 (± 0.040)
Plum	9.49 ± 0.01	71	0.121 ± 0.10	0.288 (± 0.035)
Black cherry	7.28 ± 0.07	84	0.259 ± 0.05	0.377 (± 0.037)

*The experiments were performed in triplicate and herein is reported the average (± SD)*.

**Table 3:** Analysis of correlation performed taking into consideration the values of the antioxidant (EC50, DPPH, FRAP, ABTS) and anti-inflammatory activity (IC50) of the oils with the fatty acid composition, using the free software environment for statistical computing and graphics R (https://www.r-project.org/).

	**Palmitic**	**Palmitoleic**	**Stearic**	**Oleic**	**Linoleic**	**Linolenic**
EC50	−0.85	0.58	−0.94	0.89	−0.81	0.45
DPPH	0.87	−0.67	0.88	−0.82	0.75	−0.36
FRAP	0.70	−0.42	0.87	−0.79	0.71	−0.61
ABTS	0.85	−0.51	0.82	−0.58	0.44	−0.35
IC50	0.54	−0.51	0.74	−0.91	0.93	−0.55

*The analysis was performed using the free software environment for statistical computing and graphics R (https://www.r-project.org/)*.

### Anti-inflammatory Activity

Protein denaturation is a process that gives rise to the loss of protein tertiary structure and secondary structure due to the intervention of external stress or compounds, such as strong acid or base, concentrated inorganic salts, organic solvents, or heat. Several biological proteins fail their biological function when they are denatured. The denaturation of tissue proteins is one of the well-documented causes of inflammation (53). Therefore, the determination of protein denaturation can be useful as a screening assay for the detection of anti-inflammatory compounds, without the use of animals (54, 55). Thus, we determined the action of the oils against the protein denaturation to evaluate their potential *in vitro* anti-inflammatory activity, assessing the oil amount for inhibiting the protein denaturation at 50% (IC50). The results are shown in Table 4. The cold-pressed seed oils of apricot, peach, cherry, plum, and black cherry protected the albumin against heat-induced denaturation. The amount of oil needed for 50% inhibition was very low, ranging between 3.29 and 4.34 μg, and the apricot seed oils seemed the most effective (IC50 = 3.29 μg). All the oils demonstrated a stronger anti-inflammatory activity than diclofenac, which was used as the control. The anti-inflammatory activity of the oils of apricot, peach, and plum resulted in high significance vs. the control, diclofenac (*p* < 0.001). The correlation between the EC50 values of EC50 and IC50 resulted in *r* = −0.33. This means that an antioxidant activity did not correspond to a similar anti-inflammatory effect. The anti-inflammatory activity of these oils confirmed previous studies that ascertained the potential anti-inflammatory property of seed oils, such as that of pumpkin seed oil (56); therefore, the test was also used by Harrabi et al. (57) to evaluate the anti-arthritic activity of milk thistle (*Silybium marianum* L.) oil. To the best of our knowledge, no papers reported the protective effect of these oils against protein denaturation. The extracts of the different parts of the *Prunus* species can potentially act in the treatment of inflammatory skin diseases (58) and could represent a complementary medicine for inflammatory bowel diseases (59). Peach oil has shown a protective effect in atherosclerosis that, as known could connect to some inflammatory processes (60) or act, as in the case of cherry oil, to have hearth protective effects against ischemia (61). Different studies confirmed the anti-inflammatory action of FAs. Polyunsaturated FAs exhibit anti-inflammatory properties in many inflammatory diseases (62). Dietary supplements of PUFA have been supposedly beneficial in the treatment of Irritable Bowel Disease (IBD), psoriasis, eczema, rheumatoid arthritis, and ulcerative colitis, in this last case reducing mucosal damage (63). Diets containing α-linolenic (ω3) or oleic (ω9) FAs rescued obese mice from insulin resistance (64). Among the seed oils, that extracted from *Ficus indica* contained palmitic acid (10.68%), linoleic acid (5.9%), oleic acid (8.16%), and β-sitosterol (24.98%). Therefore, in carrageenan-induced rats, this seed oil inhibited the edema reducing concurrently prostaglandin concentrations in exudates significantly (65). In our experiments, although the presence of the oleic acid (resulting usually the most abundant FA of the *Prunus* oils) seemed to inhibit the antioxidant activity of the oils (*r* = 0.89, Table 3), on the contrary, it beneficially acted to preserve the protein denaturation (*r* = −0.91, Table 3). This is of particular importance. It is ascertained that oleic acid has a valuable effect on the gut microbiota against ulcerative colitis (66). Therefore, fruits rich in unsaturated oils, such as avocado, can exhibit an anti-inflammatory effect if present in a daily diet model (67). The lipid fraction of the fruit of St Lucy cherry (*P. mahaleb* L.), rich in oleic and linoleic acids, exhibited prophylactic anti-inflammatory activity (68). Linolenic acid (*r* = −0.55, Table 3) and palmitoleic acid (*r* = −0.51, Table 3) concurred to beneficially affect the anti-inflammatory activity of the oils, although present in little amount. Thus, the oils of apricot, peach, plum, cherry, and black cherry could be nutritionally considered as a potential source of edible seed oils, with health-promoting properties, having antioxidant and potential anti-inflammatory activities, and exploited more by the food industry.

**Table 4:** Potential anti-inflammatory activity exhibited by the cold-pressed seed oils of apricot, peach, cherry, black cherry, and plum, assessed through the capacity of the oils to inhibit the denaturation of the bovine serum albumin (BSA).

	**IC 50 (± SD) (μg of oil need to inhibit at 50% the bovine serum albumin denaturation)**
Apricot	3.292^a^ (± 0.206)
Peach	3.428^a^ (± 0.415)
Cherry	4.339 (± 0.403)
Black cherry	3.864^b^ (± 0.47)
Plum	3.379^a^ (± 0.997)
Diclofenac	5.475 (± 0.32)

*The data are reported as IC50 (± SD) representing the amount of the oils (μg) need to inhibit the BSA denaturation at 50%*.

*The experiments were performed in triplicate and herein is reported the average (± SD)*.

a *: p < 0.1*;

b *: p < 0.01 compared with the diclofenac used as control (ANOVA followed by Dunnett's multiple comparison test)*.

### Antibacterial and Antibiofilm Activity

In our experiments, the antibacterial activity of the cold-pressed seed oils of the species of *Prunus* was tested against two Gram-positive bacteria, *L. monocytogenes* and *S. aureus*, and the Gram-negative bacteria *P. aeruginosa, E. coli*, and *A. baumannii*. The minimal inhibitory concentration (MIC) of the cold-pressed seed oils of *Prunus* needed to block the growth of the five species of bacteria, was assessed through the resazurin test. The results are reported in Table 5. In the case of black cherry oil vs. *A. baumanii* and *L. monocytogenes*, plum oil vs. *E. coli* and *Ps. aeruginosa*, cherry oil vs. *Ps. aeruginosa*, the activity resulted in high significance (*p* < 0.1 and *p* < 0.01). In some cases, our results demonstrated an antibacterial activity considerably superior to that of *P. persica* oil found by Bhandari et al. against *E. coli* (69). The black cherry oil exhibited greater antibacterial activity against *L. monocytogenes* than that reported by Kazempour-Samak about sour cherry seed oil, wherein the MIC against *L. monocytogenes* resulted in 100 mg/ml (70). The antibacterial capacity exhibited by all the oils (MIC not superior to 30.5 μg) confirmed the possibility of using such oils as natural preserving agents, also for the preservation of perishable food such as meat (71), and used for the formulation of new packaging material suitable for food preservation (72). The tests carried out concerned the determination of the MIC (Table 5) and subsequently, the analysis of the effect that the different oils could exercise on the capacity of bacteria to act on the biofilm and the metabolism of the bacterial cells present within the biofilm (Tables 6, 7). The ability of oils to block bacterial growth did not depend on their different cell structure, although Gram-positive and Gram-negative bacteria have a diverse resistance/sensitivity to antibiotics, also corresponding to the different cell wall structures (73). We also evaluated the aptitude of the oils to inhibit the biofilm and affect the metabolism of the cells present within the biofilm. Although several data demonstrate the antimicrobial potential of the plant species, still, a lot should be done taking into consideration both explored and unexplored plant species (74), mainly taking into consideration their by-products that could potentially be useful as complementary support in human nutrition and for the treatment of different diseases (8, 22, 75). Thus, the investigation that was undertaken in our study also tried to highlight the anti-biofilm potential of these oils as well as their action on microbial cell metabolism, which was not reported earlier, although several studies ascertained the antimicrobial properties of the extracts of the plant of genus *Prunus* (69, 76–78). Through the colorimetric analysis with crystal violet, we assessed the capacity of the cold-pressed seed oils of apricot, peach, cherry, plum, and black cherry to affect the biofilm formation (Table 6) and to act against the mature biofilm (Table 7), and we quantified the concurrent metabolism of microbial cells by colorimetric analysis with MTT. We used two concentrations of each sample, 9 and 18 μg/ml, both below what is needed to inhibit the microbial growth. *Staphylococcus aureus* turned out to be the most metabolically sensitive microbial strain to the action of *Prunus* seed oils: in fact, all the oils inhibited the metabolism of its cells, with inhibition ranging between 21.6% (caused by 9 μg/ml of peach oil) and 75.54% (determined by the presence of 18 μg/ ml of apricot oil, see Table 6). The oils managed to exert an inhibitory action on the biofilm of the microbial strain, although not with the same force exerted on the cellular metabolism. Comparing the data of MTT and crystal violet, we observed that overall, the oils of cherry seeds and plum (Table 6) acted with the same force both against the formation of the biofilm and against the metabolism of the microbial cells trapped and protected within the biofilm. In any case, where the oils failed to exert a marked inhibitory effect on the biofilm, they were able to act on cellular metabolism and vice versa. *Acinetobacter baumannii* was sensitive to black cherry oil, concerning both the formation of the biofilm and the action on the cellular metabolism within the biofilm (inhibition equal to 60.62 and 42.54%, respectively, Table 6). The other oils proved to be capable of acting, above all, on cellular metabolism. In this context, apricot oil acted on the cellular metabolism of *A. baumannii* with an inhibitory efficacy three times higher than the action exerted on the formation of the biofilm (with inhibition percentages equal to 75.07 and 25.47%, respectively, Table 6). Cherry oil exhibited an inhibiting action on the cellular metabolism of *A. baumannii* which was twice that exerted on the biofilm (41.59 and 20.07%, respectively, Table 6). Peach and plum oils, on the other hand, while acting (albeit not so markedly) on the biofilm, were incapable to act on the cellular metabolism of *A. baumannii*, so much so that even at the maximum concentration of the oil used in the test, the two oils were completely ineffective on the metabolism of this bacterium. *Escherichia coli* was sensitive to all the oils, which were able to inhibit the formation of biofilm, with inhibition percentages that, at the highest concentration of the oils tested, were between 22.10% (caused by peach seed oil) and 57.72% (determined by the presence of plum seed oil). Only black cherry oil, which in any case caused inhibition of the biofilm formation equal to 36.4%, had no inhibitory effect on bacterial metabolism. The inhibitory action shown by the oils against this microorganism can be of great consequence. In the last decades, *A. baumannii* emerged as an important nosocomial pathogen able to grow at various temperatures and pH conditions (79, 80). The versatile organism can persist in either moist or dry conditions in the hospital environment, thereby contributing to transmission (81). This hardiness, combined with its intrinsic resistance to many antimicrobial agents, contributes to the fitness of the microorganism and enables it to spread in the hospital setting. The oils we analyzed were effective in inhibiting the formation of the biofilm of *L. monocytogenes*, with a percentage of inhibition equal to 71.40% (value recorded by plum seed oil at a concentration of 18 μg/ ml). In this case, cherry oil was equally effective both in inhibiting the formation of the *L. monocytogenes* biofilm (46.18%) and above all in acting on its metabolism (56.94%). The capacity of the oils, such as the oil of plum, to inhibit the biofilm produced by *L. monocytogenes* could also be important in the food industry, as this is one of the most dangerous food pathogens to fight with chemicals (82) that often, once ingested, can give rise to harmful compounds (83). Thus, these oils, possessing antimicrobial properties and capable to be concurrently good antioxidants, could be used to prolong and preserve the shelf life of different foods, simultaneously avoiding events of oxidation that lead to a deterioration in the quality of the product. All the oils proved to be able to act against *E. coli, L. monocytogenes*, and *P. aeruginosa*, to avoid those processes that lead to the formation of the biofilm ab origin. The experiments highlighted that black cherry seed oil, although ineffective on bacterial metabolism, inhibited, at a concentration of 18 μg/ml, the formation of biofilm by *P. aeruginosa*, with a percentage of inhibitory action equal to 57.41%; the percentage of inhibition was already equal to 56.51% when we used 9 μg/ml of that oil. The other oils were able to act on the metabolism of *P. aeruginosa*, a very virulent wound pathogen commonly present in poly-microbial biofilms found in chronic wounds (84). The infections provoked by *P. aeruginosa* are particularly difficult to cure due to the structure of its biofilm matrix that inhibits the antibiotics to penetrate the biofilm (85, 86). We also provided an evaluation of the potential effect of these oils on mature biofilms by adding the same concentration of the oils after 24 h of bacterial growth, when the microorganisms have already formed the biofilm, and the metabolism of the bacterial cells is different with respect to the initial state. The results are shown in Table 7. *Staphylococcus aureus* was always sensitive to the inhibitory action of the various oils. Apricot oil—already at a concentration of 9 μg/ml—was able to exert a much more marked effect on the mature biofilm of this microorganism than it did when we added it at the start of microbial growth. Furthermore, if this oil at the beginning of microbial growth determined a negligible inhibitory effect (3.88%, Table 6), the same concentration, added after 24 h of growth, was able to act on the biofilm in a far superior way, determining an inhibition equal to 40.90%, which increased to 48.22% using 18 μg/ml of the apricot seed oil. Cherry oil (72.88 vs. 52.32% inhibition observed on immature biofilm), peach oil (whose inhibitory action increased from 32.8% on immature biofilm to 66.11% on mature biofilm), and plum oil (with a percentage of inhibition that progressed from 46.06 to 52.72%) showed similar behavior. However, in the case of the black cherry oil, we observed a slight decrease in the inhibitory action, which fell from 17.70 to 10.89%. All the oils acted more effectively on the mature biofilm of *P. aeruginosa*, or in any case (as was observed in the case of black cherry oil) kept its effectiveness intact. Therefore, these oils proved to be capable of counteracting the formation of the biofilm ab origin and/or of acting on the mature biofilm, often even more effectively, while losing efficacy in their inhibitory action on the cellular metabolism of both *S. aureus* both of *P. aeruginosa*. In the case of *A. baumannii*, we noted that some oils, in particular, peach and plum, were ineffective on cell metabolism when added at the beginning of microbial growth and managed to act on the metabolism of the cells present within the mature biofilm. On the other hand apricot oil, while counteracting the mature biofilm (27.22% inhibition), proved to be completely ineffective in countering the metabolism of the cells trapped in them. The action of the oils on the mature biofilm of *L. monocytogenes* was quite efficient. Plum oil, although its effectiveness decreased by 30%, from 71.40 to 40.60% on the mature biofilm, it doubled in efficiency passing from 12.26 to 25.06%. Similarly, black cherry oil was more effective in countering the metabolism of the *L. monocytogenes* cells present in the mature biofilm, so much so that we noted an inhibition equal to 30.80%. The various oils exhibited also inhibitory activity on the mature biofilm formed by *E. coli*, although in some cases (for instance in tests carried out with plum oil), such effectiveness decreased by 50%, passing from 57.52% inhibition on immature biofilm to 29.04% inhibition when added after 24 h of microbial growth. In addition, the oils, albeit in different ways, also acted on the metabolism of the *E. coli* cells present within the mature biofilm.

**Table 5:** Minimal inhibitory concentration (MIC, μg/ml) of the different cold-pressed seed oils of apricot, cherry, peach, plum, and black cherry needed to block the growth of the five bacterial strains, evaluated by the resazurin test.

	**AB**	**EC**	**LM**	**PA**	**SA**
Apricot oil	22.5^c^ (± 2.0)	26.5 (± 2.0)	27^d^ (± 3.0)	27^c^ (± 2.0)	27^d^ (± 2.0)
Peach oil	30.5 (± 2.0)	26.5 (± 3.0)	26^d^ (± 2.0)	26^d^ (± 3.0)	26^d^ (± 2.0)
Cherry oil	24.5^b^ (± 3.0)	26.5 (± 2.0)	26.5^d^ (± 2.0)	30.5^a^ (± 5.0)	22^d^ (± 2.0)
Plum oil	30 (± 2.0)	30^a^ (± 2.0)	24^d^ (± 3.0)	30^a^ (± 3.0)	24^d^ (± 3.0)
Black cherry oil	25.5^a^ (± 2.0)	27 (± 2.0)	31.5^b^ (± 2.0)	22.5^d^ (± 2.0)	27^d^ (± 2.0)
Tetracycline	21 (± 1.0)	24 (± 3.0)	39 (± 2.0)	36 (± 3.0)	38 (± 2.0)

*Results are expressed as the mean of three experiments ± SD*.

a *: p < 0.1*;

b *: p < 0.01*;

c *: p < 0.001*;

d *: p < 0.0001 compared with the tetracycline used as control (ANOVA followed by Dunnett's multiple comparison test). AB, A. baumannii; EC, E. coli; LM, L. monocytogenes; PA, P. aeruginosa; SA, S. aureus*.

**Table 6:** Inhibitory activity of the cold-pressed seed oils of apricot, cherry, peach, plum, and black cherry, tested at 9 and 18 μg/ml, on the biofilm formation capacity of the five pathogenic strains.

		**MTT**					**CV**				
	**Conc**	**AB**	**EC**	**LM**	**PA**	**SA**	**AB**	**EC**	**LM**	**PA**	**SA**
Apricot oil	9 μg/ml	55.38^d^(± 2.91)	2.61^d^ (± 0.12)	27.97^d^(± 1.99)	0 (± 0)	49.66^d^(± 1.28)	20.34^d^ (± 4.24)	20.69^d^(± 2.61)	31.50^d^ (± 5.12)	0(± 0)	3.88^a^ (± 1.07)
Apricot oil	18 μg/ml	75.07^d^(± 1.77)	27.72^d^ (± 2.96)	63.00^d^(± 1.64)	61.72^d^ (± 1.05)	75.54^d^(± 1.92)	25.47^d^ (± 5.46)	27.72^d^(± 2.79)	36.52^d^ (± 2.65)	21.41^d^(± 1.05)	26.65^d^ (± 1.06)
Cherry oil	9 μg/ml	15.33^d^(± 2.93)	32.46^d^ (± 2.21)	0(± 0)	0 (± 0)	46.23^d^(± 1.91)	15.57^d^ (± 1.41)	21.66^d^(± 1.32)	36.23^d^ (± 3.34)	0(± 0)	32.25^d^ (± 2.21)
Cherry oil	18 μg/ml	41.59^d^(± 1.94)	45.15^d^ (± 1.22)	56.94^d^(± 1.43)	51.18^d^ (± 3.94)	55.66^d^(± 1.37)	20.07^d^ (± 1.89)	23.41^d^(± 1.60)	46.18^d^ (± 2.94)	22.14^d^(± 1.48)	52.32^d^ (± 3.62)
Peach oil	9 μg/ml	0(± 0)	38.82^d^ (± 2.16)	35.88^d^(± 1.89)	24.41^d^ (± 2.16)	21.16^d^(± 1.32)	9.50^d^ (± 1.50)	17.32^d^(± 1.09)	37.80^d^ (± 2.76)	0(± 0)	10.57^d^ (± 1.12)
Peach oil	18 μg/ml	0(± 0)	51.38^d^ (± 1.77)	40.33^d^(± 1.05)	50.74^d^ (± 1.16)	53.85^d^ (± 2.70)	12.42^d^ (± 1.55)	22.10^d^(± 1.60)	42.08^d^ (± 1.48)	10.46^d^(± 1.69)	32.58^d^ (± 2.98)
Plum oil	9 μg/ml	0(± 0)	43.64^d^ (± 1.22)	7.91^d^(± 0.57)	24.43^d^ (± 1.47)	39.55^d^(± 0.95)	12.96^d^ (± 1.16)	9.93^d^(± 1.39)	26.25^d^ (± 1.83)	8.03^d^(± 0.98)	24.39^d^ (± 1.96)
Plum oil	18 μg/ml	0(± 0)	44.60^d^ (± 0.53)	12.26^d^(± 2.00)	56.15^d^ (± 0.48)	68.60^d^(± 1.79)	24.03^d^ (± 1.26)	57.72^d^(± 1.59)	71.40^d^ (± 2.55)	16.52^d^(± 1.10)	46.06^d^ (± 2.79)
Black cherry oil	9 μg/ml	0(± 0)	0 (± 0)	0(± 0)	0 (± 0)	25.33^d^(± 1.47)	20.73^d^ (± 1.18)	30.41^d^(± 3.25)	0 (± 0)	56.51^d^(± 4.82)	10.84^d^ (± 0.44)
Black cherry oil	18 μ g/ml	42.54(± 0.44)	0 (± 0)	0(± 0)	0 (± 0)	69.44^d^(± 4.42)	60.62^d^ (± 3.28)	36.4^d^(± 2.23)	25.25^d^ (± 2.99)	57.41^d^(± 4.58)	17.70^d^ (± 1.58)

*Results are expressed as percentages (average ± SD) and calculated assuming the control (untreated bacteria, for which we assumed an inhibitory value= zero)*.

a *: p < 0.1*;

*^b^: p < 0.01*;

*^c^: p < 0.001*;

d *: p < 0.0001 compared with the control (ANOVA followed by Dunnett's multiple comparison test). AB, A. baumannii; EC, E. coli; LM, L. monocytogenes; PA, P. aeruginosa; SA, S. aureus. MTT, data of the inhibitory action exhibited by the oils on bacterial metabolism, evaluated through the MTT test; CV, data of the inhibitory action exhibited by the oils on the biofilm, evaluated through the Cristal violet assay*.

**Table 7:** Inhibitory activity of the cold-pressed oils of apricot, cherry, peach, plum, and black cherry, tested at 9 μg/ml and 18 μg/ml, on the inhibitory capacity on mature biofilm by five pathogenic bacteria.

		**MTT**					**CV**				
	**Conc**	**AB**	**EC**	**LM**	**PA**	**SA**	**AB**	**EC**	**LM**	**PA**	**SA**
Apricot oil	9 μg/ml	0(± 0)	0 (± 0)	0(± 0)	0 (± 0)	0(± 0)	19.82^d^ (± 4.66)	24.85^d^(± 2.27)	1.15 (± 0.55)	48.73^d^(± 0.65)	40.90^d^ (± 2.09)
Apricot oil	18 μg/ml	0(± 0)	30.59^d^ (± 1.06)	0(± 0)	0 (± 0)	40.82^d^(± 0.98)	27.22^d^ (± 2.99)	27.44^d^(± 1.73)	33.68^d^ (± 4.51)	52.38^d^(± 4.56)	48.22^d^ (± 3.31)
Cherry oil	9 μg/ml	0(± 0)	2.84 (± 0.20	0(± 0)	7.93^d^ (± 1.56)	0(± 0)	6.26^c^ (± 1.19)	24.33^d^(± 2.07)	37.32^d^ (± 2.42)	33.00^d^(± 1.19)	12.55^d^ (± 1.03)
Cherry oil	18 μg/ml	0.50(± 0.02)	22.61^d^ (± 0.55)	30.33^d^(± 1.51)	38.71^d^ (± 1.15)	32.46^d^(± 0.40)	27.40^d^ (± 1.90)	25.71^d^(± 2.19)	47.02^d^ (± 2.62)	55.33^d^(± 4.15)	72.88^d^ (± 3.62)
Peach oil	9 μg/ml	25.48^d^(± 0.51)	31.29^d^ (± 0.97)	0(± 0)	20.41^d^ (± 1.09)	8.23^d^(± 1.19)	13.92^d^ (± 6.13)	17.95^d^(± 1.19)	11.96^d^ (± 1.28)	0(± 0)	50.10^d^ (± 2.09)
Peach oil	18 μg/ml	26.26^d^(± 0.73)	44.21^d^ (± 0.92)	0(± 0)	36.80^d^ (± 1.99)	18.06^d^(± 0.59)	29.52^d^ (± 1.70)	25.80^d^(± 1.64)	44.11^d^ (± 2.06)	56.36^d^(± 1.69)	66.11^d^ (± 2.06)
Plum oil	9 μg/ml	0(± 0)	0 (± 0)	0.83(± 0.15)	6.38^c^ (± 0.93)	0(± 0)	14.63^d^ (± 1.40)	24.94^d^(± 1.92)	15.02^d^ (± 1.19)	34.26^d^(± 1.56)	49.58^d^ (± 2.04)
Plum oil	18 μg/ml	12.60^d^(± 0.94)	42.64^d^ (± 3.08)	25.06^d^(± 0.40)	16.69^d^ (± 1.59)	6.83^d^(± 0.28)	17.44^d^ (± 1.41)	29.04^d^(± 1.27)	40.60 (± 2.31)	49.97^d^(± 0.85)	52.72^d^ (± 2.67)
Black cherry oil	9 μg/ml	19.96^d^(± 1.16)	0 (± 0)	0(± 0)	0 (± 0)	0(± 0)	0 (± 0)	0(± 0)	4.91^b^ (± 0.70)	32.09^d^(± 1.69)	0 (± 0)
Black cherry oil	18 μg/ml	44.36^d^(± 0.88)	15.11^d^ (± 0.62)	30.80^d^(± 2.60)	0 (± 0)	21.51^d^(± 1.23)	61.54^d^ (± 0.79)	31.03^d^(± 2.76)	20.44^d^ (± 1.78)	56.48^d^(± 2.44)	10.89^d^ (± 0.88)

*Results are expressed as percentages (average ±SD) and calculated assuming the control (untreated bacteria, for which an inhibitory value= zero was assumed)*.

*^a^: p < 0.1*;

b *: p < 0.01*;

c *: p < 0.001*;

d *: p < 0.0001 compared with the control (ANOVA followed by Dunnett's multiple comparison test). AB, A. baumannii; EC, E. coli; LM, L. monocytogenes; PA, P. aeruginosa; SA, S. aureus; MTT, data of the inhibitory action exhibited by the oils on bacterial metabolism, evaluated through the MTT test; CV, data of the inhibitory action exhibited by the oils on the biofilm, evaluated through the Cristal violet assay*.

## Conclusion

The five oils of *Prunus* analyzed could provide interesting insights and potential applicability, opening new opportunities for their use. They could represent functional ingredients in the manufacturing of different types of food—for instance, baked foods—as a replacement for butter or hydrogenated fats, too often present in recipes. Biscuits, snacks, and creams for snacks, containing these types of oils would represent foods with nutritional and health value suitable for consumers. The anti-inflammatory activity exhibited by the oils, with results better than that of the conventional diclofenac, also have potential applicability as natural anti-inflammatory agents through their regular presence in a dietary plan. This could avoid, as much as possible, the use of synthetic anti-inflammatories, with long-term damage to the organism. The inhibitory action exhibited by these oils both against the biofilm formation and against the mature biofilm, allowed us to glimpse new applicative perspectives in the health and food fields. These oils could represent potential natural combat weapons toward the onset of infections caused by pathogens that the biofilm makes more difficult to eradicate.

## Data Availability Statement

The original contributions presented in the study are included in the article, further inquiries can be directed to the corresponding author/s.

## Author Contributions

FN: research plan, microbial analysis, data elaboration, writing, and supervision. FF and MNO: biochemical analysis. Ad'A: data processing. GA: biochemical analysis and data processing. VD: biochemical analysis and research plan. JA-Z and RC: research plan. All authors contributed to the article and approved the submitted version.

## Funding

This work was partly funded by the National Research Council of Italy (CNR) project Nutrizione, Alimentazione & Invecchiamento Attivo (NUTR-AGE, FOE-2019, DSB. AD004. 271).

## Conflict of Interest

The authors declare that the research was conducted in the absence of any commercial or financial relationships that could be construed as a potential conflict of interest.

## Publisher's Note

All claims expressed in this article are solely those of the authors and do not necessarily represent those of their affiliated organizations, or those of the publisher, the editors and the reviewers. Any product that may be evaluated in this article, or claim that may be made by its manufacturer, is not guaranteed or endorsed by the publisher.
